# Low Digit Ratio 2D∶4D in Alcohol Dependent Patients

**DOI:** 10.1371/journal.pone.0019332

**Published:** 2011-04-25

**Authors:** Johannes Kornhuber, Gabriele Erhard, Bernd Lenz, Thomas Kraus, Wolfgang Sperling, Kristina Bayerlein, Teresa Biermann, Christina Stoessel

**Affiliations:** 1 Department of Psychiatry and Psychotherapy, Friedrich-Alexander University of Erlangen-Nuremberg, Erlangen, Germany; 2 Department of Psychiatry and Psychotherapy, Frankenalbklinik Engelthal, Engelthal, Germany; Baylor College of Medicine, United States of America

## Abstract

The ratio of the lengths of the second and fourth finger (2D∶4D) has been described as reflecting the degree of prenatal androgen exposure in humans. 2D∶4D is smaller for males than females and is associated with traits such as left-handedness, physical aggression, attention-deficit-hyperactivity disorder and a genetic polymorphism of the androgen receptor. All of these traits are known to be correlated to the vulnerability for alcohol dependency. We therefore hypothesized low 2D∶4D in patients with alcohol dependency. In the present study on 131 patients suffering from alcohol dependency and 185 healthy volunteers, we found that alcohol dependent patients had smaller 2D∶4D ratios compared to controls with preserved sexual dimorphism but with reduced right-left differences. The detection of alcohol dependency based on 2D∶4D ratios was most accurate using the right hand of males (ROC-analysis: AUC 0.725, sensitivity 0.667, specificity 0.723). These findings provide novel insights into the role of prenatal androgen exposure in the development of alcohol dependency and for the use of 2D∶4D as a possible trait marker in identifying patients with alcohol dependency.

## Introduction

The lengths of the second digit (2D) and fourth digit (4D) and their ratio have received attention because of sex differences [1]. The ratio of 2D to 4D is smaller for males than females. Those differences are generally larger for the right hand than for the left in humans [2]–[4]. Sexual dimorphism is already seen in the 9^th^ week of the foetal period [3]. Low 2D∶4D values probably result from high prenatal testosterone exposure [1], [4], [5]. Evidence supporting this hypothesis has recently been reviewed [6], but for an alternative critical view see Forstmeier et al., 2010 [7].

Several of the phenomena that are observed in persons with low 2D∶4D ratio have also been found in patients with alcohol dependency. Men have lower 2D∶4D ratios and consume more alcohol [8], [9]. Left-handedness is associated with altered 2D∶4D measures [10]–[13], and is also more frequent in patients with alcohol dependency [14] (however, see also [15]). Low 2D∶4D is associated with attention-deficit-hyperactivity disorder [16], which is also associated with alcohol dependency [17]. Low 2D∶4D correlates with psychological traits such as physical aggression, novelty seeking and higher dominance [18]–[20]. These features have also been reported as predictors of substance abuse [8], [21], [22]. Finally, a CAGn trinucleotide repeat within the coding region of the androgen receptor has been shown to correlate with variation in 2D∶4D ratio. Thus, men with shorter alleles ( = more sensitive androgen receptors) possess more masculinized right hand 2D∶4D ratios relative to their left 2D∶4D, and this correlation was driven by a positive association between right 2D∶4D and CAGn [23]. However, while Hurd et al. [18] found a similar association between right 2D∶4D-left 2D∶4D (Dr-l) and CAGn, in this case the correlation was driven by a positive association between left 2D∶4D and CAGn. This polymorphism is associated with craving for alcohol of male patients during withdrawal [24]. We therefore hypothesized that patients with alcohol dependency have been exposed to high prenatal testosterone levels, as indicated by the proxy 2D∶4D.

## Methods

This study is part of the FLIP-project (Finger-Length In Psychiatry). Between February 2009 and March 2010 we recruited 131 patients (87 males and 44 females; age between 24 and 77 years, median age 46.4) who were treated as inpatients in the Department of Psychiatry in the Frankenalbklinik Engelthal. All patients suffered from alcohol dependency according to ICD-10-criteria. Controls consisted of 185 volunteers (83 males and 102 females; age between 19 and 64 years; median age 37.5) recruited predominantly from among employees of the University of Erlangen-Nuremberg via direct personal contact. Written informed consent was obtained after complete description of the study to the subjects. The study was approved by the local Ethics Committee (Ethik-Kommission der Medizinischen Fakultät der Friedrich-Alexander Universität Erlangen-Nürnberg).

Scanning was conducted prior to examining or analyzing questionnaires scores. A HP scan-jet G4050 was used to scan participants' hands. To increase accuracy, small marks were drawn on the basal creases of the index and ring fingers before scanning. Both hands were scanned at the same time, palms down. We used the GNU Image Manipulation Program (GIMP, version 2.6.8 p1; www.gimp.org) to measure the index (2D) and ring (4D) fingers from the hand scans. This technique provides good reliability [19]. The total length of the second and fourth digit of the fight and left hands was measured from the middle of the basal crease to the tip of the finger and was determined in units of pixels, using the GIMP “measure” tool. The measurements were performed by three independent persons, first by one experimenter (GE) and also by two untrained persons blind to the hypothesis and blind to the diagnostic category. Each of the three persons had to perform 1264 measurements (131 patients and 185 controls, two digits, two hands). Mean values of the three measurements were calculated for the second and fourth digit.

The reliability of the three measurements was calculated for each finger separately for the right and left hand using the two-way random intra-class correlation coefficient (ICC) [25]. ICCs were also calculated for 2D∶4D ratios and Dr-l values. Deviation from normal distribution was tested by Kolmogorof-Smirnov-Test. 2D∶4D ratios of the subjects were analyzed by a 2 (diagnosis)×2 (sex)×2 (hand) factorial ANCOVA with repeated measurement on the factor hand and age as covariate. Dr-l values were analyzed by a 2 (diagnosis)×2 (sex) factorial ANCOVA with age as covariate. The effect size of the main factors or interactions was estimated by partial η^2^-values. Partial η^2^-values, in contrast to classical η^2^-values, are defined as the proportion of total variation attributable to the experimental factor partialling out other factors from the total nonerror variation [26]. To estimate the value of 2D∶4D ratio as a diagnostic test for discrimination of alcohol dependent patients from controls, we used a ROC-analysis to calculate AUC values (ranging from 0.5 = random prediction to 1 = perfect prediction), as well as sensitivity and specificity at the Youden-point (the point on the ROC-curve, where the sum of sensitivity and specificity is maximized). A p-value<0.05 (two-tailed) was regarded as significant. All statistical analyses were computed using PASW (Version 18, Chicago, Illinois).

## Results

Reliability of the three raters was high for both the right hand (2D: ICC = 1.000; 4D: ICC = 0.998; 2D∶4D: ICC = 0.988) and the left hand (2D: ICC = 0.991; 4D: ICC = 0.998; 2D∶4D: ICC = 0.951). Reliability of the Dr-l values were also high (ICC = 0.898). The mean 2D∶4D and Dr-l values are presented in Table 1. 2D∶4D and Dr-l values did not deviate from normal distribution.

**Table 1:** 2D∶4D and Dr-l values in patients and controls.

	Patients		Controls	
	Males (n = 87)	Females (n = 44)	Males (n = 83)	Females (n = 102)
2D∶4D right hand	0.952±0.031	0.967±0.030	0.976±0.029	0.983±0.032
2D∶4D left hand	0.949±0.034	0.967±0.031	0.967±0.029	0.976±0.030
Dr-l	0.0029±0.0278	0.0003±0.0218	0.0086±0.0251	0.0067±0.0241

Values are means ± standard deviation (SD). Dr-l = right 2D∶4D - left 2D∶4D.

**Figure 1:**
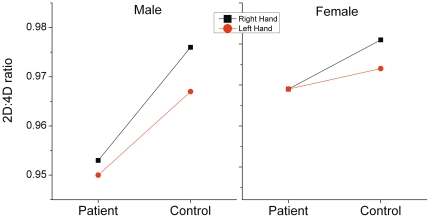
Interaction line plots. A 2 (diagnosis)×2 (sex)×2 (hand) factorial ANCOVA with repeated measurement on the factor hand with age as a covariate shows a reduced 2D∶4D ratio in alcohol dependent patients with preserved sexual dimorphism and reduced right-left difference.

2D∶4D ratio was most associated, in descending order, with diagnosis (F = 16.0, df = 1, p<0.001, partial η^2^ = 0.049), sex (F = 13.2, df = 1, p<0.001, partial η^2^ = 0.041), hand*diagnosis-interaction (F = 4.4, df = 1, p = 0.038, partial η^2^ = 0.014) and age (F = 4.2, df = 1, p = 0.041, partial η^2^ = 0.013). There were no further significant main or interaction effects. The interaction line plots for 2D∶4D ratios for male and female subjects are shown in figure 1. Diagnosis was significantly associated with Dr-l values (F = 4.4, df = 1, p = 0.038, partial η^2^ = 0.014) with no further main or interaction effects. Together, these results indicate that alcohol dependent patients have smaller 2D∶4D ratios with preserved sexual dimorphism but with reduced right-left differences. The ROC analysis indicates highest diagnostic accuracy of the 2D∶4D ratio of the right hand in males (AUC 0.725, sensitivity 0.667, specificity 0.723), followed by males' left hand (AUC 0.667, sensitivity 0.529, specificity 0.759), females' right hand (AUC 0.650, sensitivity 0.864, specificity 0.392) and females' left hand (AUC 0.570, sensitivity 0.886, specificity 0.265).

## Discussion

This is the first study investigating prenatal testosterone exposure, as assessed by the proxy 2D∶4D, in alcohol dependent patients. Patients with alcohol dependency have low 2D∶4D ratios with preserved sexual dimorphism and reduced right-left differences. These findings are remarkable for several reasons. (1) As 2D∶4D is fixed before birth [3] and remains relatively constant during life, 2D∶4D is a novel and easily-accessible trait marker for alcohol dependency. (2) The low 2D∶4D ratio in alcohol dependent patients suggests that several phenomena that have largely been investigated independently before share a common pathophysiological basis. These phenomena include a higher life-time risk of males to develop alcohol dependency, increased prevalence of left-handedness, higher comorbidity of attention-deficit-hyperactivity disorder, higher prevalence of personality traits such as aggression, novelty seeking and higher dominance, and shorter CAGn trinucleotide repeat alleles of the androgen receptor. Sex steroids have an important organizing effect on the brain during the foetal period with subsequent long-term effects on behaviour [6]. There is considerable evidence suggesting that prenatal testosterone exposure causes masculinization of physiology, anatomy, and behaviour. The findings presented here provide a novel insight into the etiopathogenesis of alcohol dependency. High prenatal testosterone exposure, as assessed by its proxy 2D∶4D, probably presents a risk factor for the development of alcohol dependency via mechanisms to be investigated. (3) It is known that right hand 2D∶4D is a better indicator of prenatal androgenization than left hand 2D∶4D [4]. Furthermore, the difference in 2D∶4D between controls and persons with congenital adrenal hyperplasia, a condition that causes excessive androgen production during gestation, is larger on the right hand than the left hand [4]. It is therefore plausible that right hand 2D∶4D is a better predictor of alcohol dependency than left hand 2D∶4D, as shown here. Low Dr-l values have been associated with high prenatal testosterone load (Manning, 2002, pp 21–22, [27]). The significantly lower Dr-l values in alcohol dependent patients shown here support the hypothesis of high prenatal testosterone load being a risk factor for later development of alcohol dependency. (4) This study shows that right hand 2D∶4D alone provides a reasonable sensitivity and specificity in the diagnosis of alcohol dependency, comparable to state markers such as GGT [28]. However, the sensitivity and specificity of 2D∶4D as a single marker is too low to support an individual diagnosis, and possessing a low 2D∶4D ratio is neither necessary nor sufficient to develop alcohol dependency. Furthermore, the diagnostic value of 2D∶4D presented here may be overoptimistic, since we used a mono-centric design and did not apply cross-validation or external validation during data analysis [29]. The diagnostic and prognostic value of 2D∶4D as a single trait marker or in combination with other trait as well as state markers has to be investigated in future studies under clinical routine conditions using internal and external validation. (5) Our results are supported by a very recent study reporting on a negative association between 2D∶4D and alcohol consumption in the general population [30].

We used a case control design without a matched sample approach. (1) There were differences in age between the patient and control groups. In agreement with Manning et al. 2010 [31], age had a marginal significant negative effect on 2D∶4D in the present study. However, alcohol dependency was clearly associated with both small 2D∶4D and small Dr-l values, even when controlling for age effects. Furthermore, Manning and Fink [30] observed a negative relationship between alcohol consumption and 2D∶4D in the general population that was also independent of age. Therefore, it is unlikely that age had an impact on the results presented here. (2) Previous studies have shown that the educational level and the academic abilities are related to 2D∶4D. For instance, 2D∶4D was positively related to the examination marks of three-year degree courses in male, but not in female university students [32]. Women in academia have lower, more masculine 2D∶4D values [33] which explains a reduced 2D∶4D gender difference in academic populations [33], [34]. Assuming that our control sample from a university hospital environment has a higher mean level of education would possibly result in reduced female 2D∶4D ratios and would thus result in an underestimation of the difference between alcohol dependent patients and controls. (3) We did not evaluate alcohol consumption in the control group. Therefore, volunteers with moderate to heavy alcohol consumption may have been part of the control group, which might also result in an underestimation of the difference in 2D∶4D between alcohol dependent patients and control persons with low alcohol consumption.

May prolonged alcohol consumption have induced changes in 2D∶4D in later life? This appears unlikely, because (1) the differences in 2D∶4D between control subjects and alcohol dependent patients observed in our study are large. (2) Furthermore, it would be hard to explain, why alcohol should have different effects on the index finger compared to the ring finger. (3) In addition, duration of alcohol use disorder (range: 0.5–44 years) was not related to 2D∶4D in the patients investigated here when controlling for age (data not shown). However, in order to definitely answer this question would require a longitudinal study with individual tracking of changes of 2D∶4D due to heavy alcohol consumption. Such data are currently not available.

Alcohol consumption of the mother may have an impact on finger length ratios of the offspring, as has been shown in rodents. Maternal alcohol consumption results in reduced testosterone levels and more female finger length ratios [35] and, therefore, does also not explain the results presented here.

In conclusion, we were able to demonstrate that alcohol dependent patients have small 2D∶4D ratios. Prenatal androgen exposure might be a missing link between several well-known findings in alcohol research. Further studies are needed to clarify the value of 2D∶4D as a diagnostic or prognostic marker for alcohol dependency.
